# Practical guide to genetic screening for inherited eye diseases

**DOI:** 10.1177/2515841420954592

**Published:** 2020-09-22

**Authors:** Cécile Méjécase, Samantha Malka, Zeyu Guan, Amy Slater, Gavin Arno, Mariya Moosajee

**Affiliations:** Institute of Ophthalmology, University College London, London, UK; Institute of Ophthalmology, University College London, London, UK; Moorfields Eye Hospital NHS Foundation Trust, London, UK; Moorfields Eye Hospital NHS Foundation Trust, London, UK; Royal Brompton and Harefield NHS Foundation Trust, London, UK; Institute of Ophthalmology, University College London, London, UK; Moorfields Eye Hospital NHS Foundation Trust, London, UK; Great Ormond Street Hospital for Children NHS Trust, London, UK; Professor, Institute of Ophthalmology, University College London, 11-43 Bath Street, London EC1V 9EL, UK; Moorfields Eye Hospital NHS Foundation Trust, London, UK; Great Ormond Street Hospital for Children NHS Trust, London, UK; The Francis Crick Institute, London, UK

**Keywords:** family planning, genetic counselling, genetic screening, inherited eye disease, next generation sequencing, whole exome sequencing and whole genome sequencing

## Abstract

Genetic eye diseases affect around one in 1000 people worldwide for which the molecular aetiology remains unknown in the majority. The identification of disease-causing gene variant(s) allows a better understanding of the disorder and its inheritance. There is now an approved retinal gene therapy for autosomal recessive *RPE65-*retinopathy, and numerous ocular gene/mutation-targeted clinical trials underway, highlighting the importance of establishing a genetic diagnosis so patients can fully access the latest research developments and treatment options. In this review, we will provide a practical guide to managing patients with these conditions including an overview of inheritance patterns, required pre- and post-test genetic counselling, different types of cytogenetic and genetic testing available, with a focus on next generation sequencing using targeted gene panels, whole exome and genome sequencing. We will expand on the pros and cons of each modality, variant interpretation and options for family planning for the patient and their family. With the advent of genomic medicine, genetic screening will soon become mainstream within all ophthalmology subspecialties for prevention of disease and provision of precision therapeutics.

## Introduction

Worldwide, approximately one in 1000 people are affected with genetic eye disease.^1^ In the United Kingdom, one in 2500 children under the age of 1 year are diagnosed as blind or severely visually impaired with an estimated 33% having a genetic basis.^2^ Genetic eye disorders affect individuals of all ages and encompass a broad spectrum of disease including developmental eye defects, corneal and retinal dystrophies, and hereditary optic neuropathies, with both nonsyndromic and syndromic forms. These rare diseases can affect all parts of the eye including the adnexa, ocular muscles, anterior chamber and posterior segment. They can be isolated, only affecting the eye, or found in association with systemic features, forming part of a syndrome. Developmental eye defects include microphthalmia, anophthalmia and ocular coloboma (MAC), anterior segment dysgenesis, congenital cataracts, primary congenital glaucoma, retinal dysplasia and optic nerve hypoplasia. Inherited retinal disorders (IRDs) are a broad group of nonprogressive and progressive sight loss conditions characterised by a retinal degeneration. These include Leber congenital amaurosis (LCA), severe early onset retinal dystrophies, congenital stationary night blindness, achromatopsia, cone and rod dystrophies, retinitis pigmentosa (RP) and macular dystrophies. Together IRDs affect 11 per 50,000 children and are the commonest cause of blindness among working age adults in the United Kingdom.^3^

All inheritance patterns are represented for hereditary eye disorders, the vast majority of cases being caused by genetic defects involving a single gene. Mutations range from single nucleotide substitutions and insertions/deletions to whole gene or chromosomal rearrangements. Although digenic and multiallelic cases have been reported,^4,5^ there remains little evidence for this being a significant cause of disease. Similarly, although environmental factors have been found to contribute to certain ocular maldevelopment phenotypes, this is a small proportion.^2^ In one UK prospective study investigating the incidence of MAC, only 2% of cases were considered due to environmental influence such as maternal alcohol use or maternal vitamin A deficiency.^6^ Pathogenic variants in the mitochondrial genome or autosomal genes encoding mitochondrial proteins may lead to mitochondrial disorders including inherited optic neuropathies.^7^ Rarer genetic causes of disease include mosaicism and uniparental iso- and hetero-disomy.^8–11^

There is considerable genetic and phenotypic heterogeneity, and in some cases it can be extremely difficult to attribute a particular disease-causing gene unless molecularly confirmed, as this also has implications for future treatments and generations. With the first approved gene therapy for the IRDs caused by biallelic variants in the *RPE65* gene,^12^ and several more genetic-based treatments emerging, it is paramount that patients have access to the appropriate genetic testing. In this review, we provide guidance on current and future practices for genetic testing of Mendelian eye disorders, how to interpret results and guide genetic counselling.

## How to identify patients who may benefit from genetic screening?

### Family history and inheritance patterns

A detailed clinical history and examination is of key importance to determine the likely aetiology of an eye disease. In suspected genetic eye disease, the clinical findings should guide which molecular investigations/genetic testing is most suitable to ascertain the possible cause. Hence, the dissection of disease features, onset, progression and severity of symptoms for all affected family members, detailed pregnancy/birth history, family history and consanguinity are key aspects. For example, an infant presenting with congenital cataract is less likely to be suffering from an inherited form if the mother developed an intrauterine infection with rubella during early pregnancy. The family history can help to determine the inheritance pattern of Mendelian disease such as autosomal dominant, autosomal recessive, X-linked or mitochondrial. Examination should involve a full ocular and systemic assessment, with accompanying investigations, such as neuroimaging or ocular ultrasound, and imaging, for example, anterior segment or retinal optical coherence tomography (OCT) and fundus autofluorescence (FAF). Any clinical features should be recorded using human phenotypic ontology (HPO) to provide a standardised form of characterisation which may guide diagnosis and management^13,14^ (Box 1). Syndromic patients with congenital eye malformations and learning difficulties are likely to have a chromosomal abnormality^15^ and may be best referred to a paediatrician or clinical geneticist for further review and investigation. Establishing a precise molecular diagnosis for any genetic eye disease can only be achieved through genetic testing and this will allow the clinician to stratify clinical risk in terms of prognosis, co-morbidities, assemble the correct multidisciplinary team and advise on possible research and clinical trials that may benefit the patient.

Box 1. **Importance of human phenotypic ontology (HPO)**

HPO was first established in 2007 to unify phenotype description reported in the Online Mendelian Inheritance in Man (OMIM) database.^16^ Over 13,000 clinical phenotype features are regrouped and described in HPO (https://hpo.jax.org/app/), each with a unique identifier, in a ‘parent-child’ structure. An example of a small part of this structure is as follows:

• HP:0000478 Abnormality of the Eye
○ HP:0012372 Abnormal eye morphology
▪ HP:0000589 Coloboma
▪ HP:0100887 Abnormality of globe size
○ HP:0012373 Abnormal eye physiology
▪ HP:0012632 Abnormal intraocular pressure
▪ HP:0000501 Glaucoma

It is important to note HPO encompasses individual clinical features which are then regrouped into one or more disease(s), it can help to study the link with other pathologies and their genetic association.^17^ To apply HPO, the most specific terms based on its definition (and not on its name) can be chosen to describe the observed clinical features, with the help of the website browser. The absence of some clinical features (i.e. investigated and not observed) can also be reported. The HPO description will be then understandable by healthcare professionals and researchers, who work in close collaboration to solve the molecular diagnosis of genetic eye disease patients. A list of HPO terms helps to identify the relevant genes/panels that need to be screened to aid diagnosis. If genetic testing reveals a variant(s) in a gene associated with a syndromic disorder, the HPO terms can help to confirm associated systemic phenotypes, expedite diagnosis and allow for the correct multidisciplinary team to be involved.

### Autosomal recessive disease

**Figure 1.**
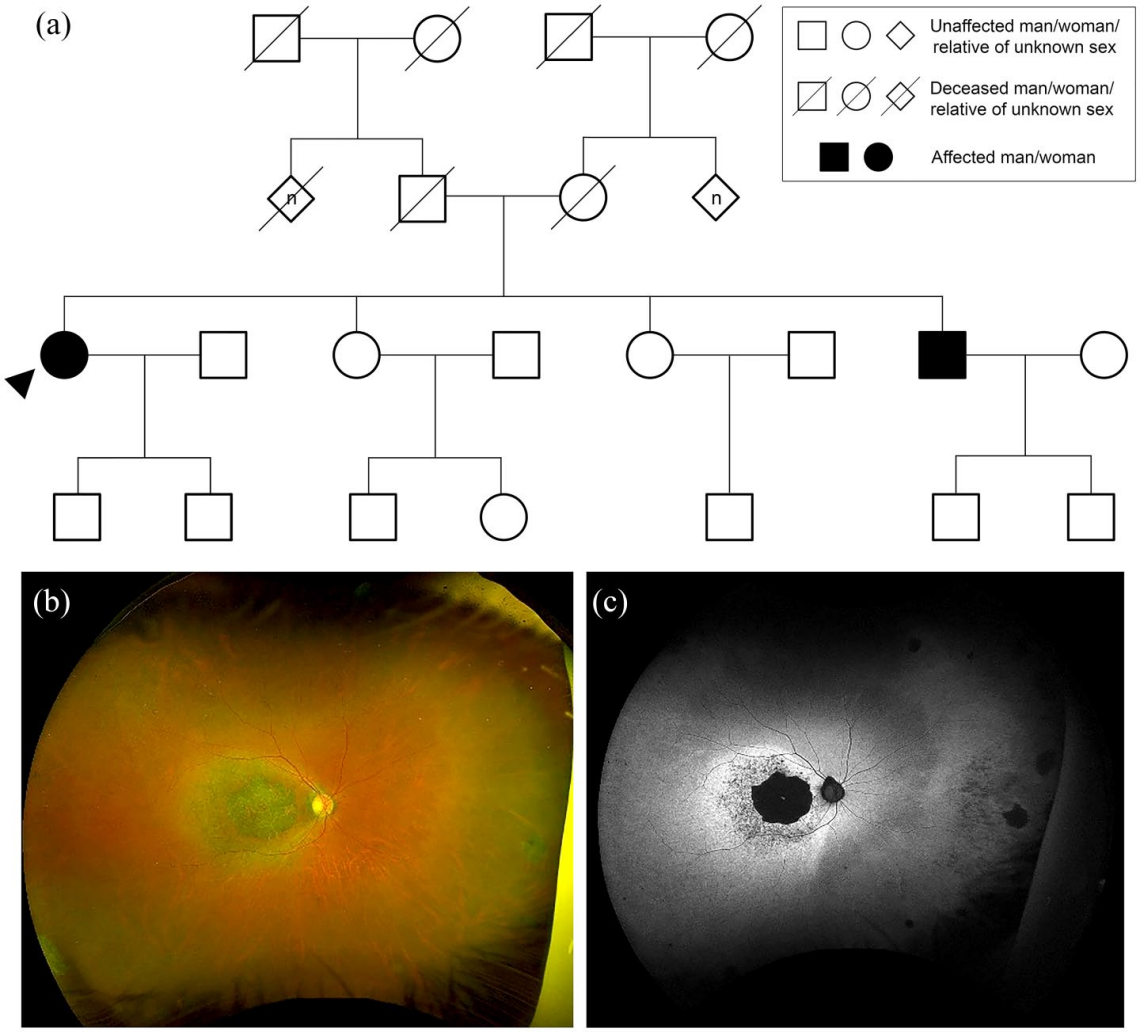
Autosomal recessive inheritance. (a) Family tree highlighting autosomal recessive cone-rod dystrophy caused by heterozygous nonsense variants in *CERKL*; c.1090C>T p.(Arg364*) and c.847C>T p.(Arg283*). Circles represent women, squares men, diamonds relatives of unknown sex. Filled forms represent affected individuals; unfilled forms unaffected individuals. Crossed forms represent deceased individuals. The ‘n’ in diamond form indicates an unknown number of relatives. (b) Widefield colour fundus photograph of the right eye of the proband aged 61 years showing macular atrophy. (c) Widefield fundus autofluorescence of the right eye showing a dense hypoautofluorescent (black) area corresponding to the area of atrophy within a surrounding hyperautofluorescent ring.

Autosomal recessive disease inheritance is defined by the presence of biallelic variants on the genes located on an autosome leading to disease. These variants range from point mutations, structural changes within a gene, or larger rearrangements/copy number variations encompassing several genes. Carriers of a single autosomal recessive pathogenic change are clinically unaffected. While there can be variability between affected individuals, autosomal recessive inheritance does not discriminate between males and females, so both sexes are equally likely to be affected (Figure 1).

Parents of an affected patient with an autosomal recessive condition will usually either be unaffected carriers (with a single monoallelic pathogenic change) or be affected with the condition themselves (with biallelic pathogenic changes). Consanguinity in parents increases the risk of autosomal recessively inherited conditions. In non-consanguineous families, autosomal recessive diseases are not often seen in multiple generations and cases will be frequently simplex. For monoallelic carrier parent couples, there is a 25% risk with each pregnancy of the child inheriting the disorder, a 25% chance of the child being unaffected and not carrying either of the pathogenic mutations and a 50% chance of the child being an unaffected carrier.

All children of individuals affected with an autosomal recessive condition will inherit one allele with a pathogenic change from their affected parent and will therefore be a carrier for the condition. If this child’s other parent is not affected, nor a carrier of a pathogenic change in the same gene, then the child will not be affected with the condition. The risk of an affected patient’s child inheriting the autosomal recessive condition is small and depends on the population frequency of the pathogenic variant. The risk of a patient with the disease passing this onto their child is less than 1%. These risks increase if there is consanguinity between the parents or a positive family history of the same condition in the unaffected parent.

There are common pathogenic autosomal recessive genes seen in inherited eye diseases. For example, Stargardt disease is a macular dystrophy with a prevalence of ~1:8000–10,000 predominantly caused by biallelic variants in *ABCA4*, which determine the onset and severity of the phenotype.^18,19^ Some disease-causing variants in the same gene can be associated with syndromic or nonsyndromic disorders, for example, *USH2A* biallelic variants can be associated with type II Usher syndrome in 85% of cases, characterised by vision and hearing loss, or nonsyndromic RP in 20% of RP cases.^20–22^ Although the majority of bilateral microphthalmia/anophthalmia cases are due to dominant monoallelic mutations, homozygous and compound heterozygous loss-of-function variants are found in *STRA6* and *RAX*.^6,23,24^

### Autosomal dominant disease

**Figure 2.**
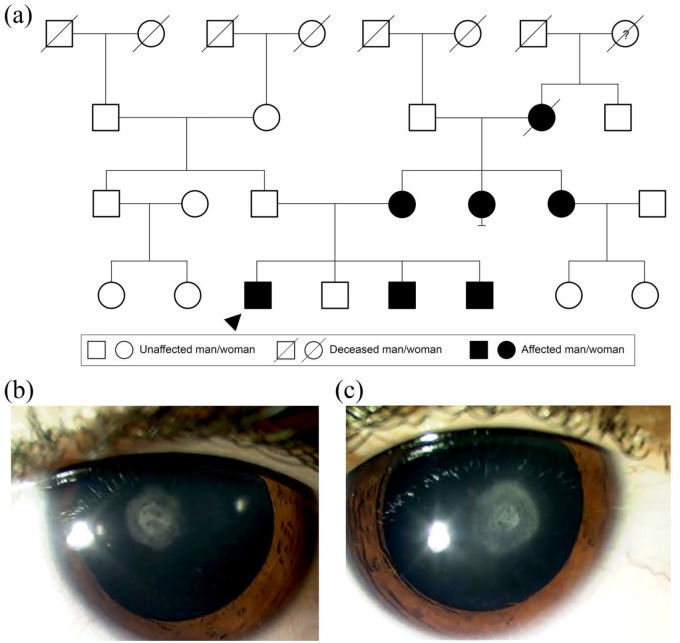
Autosomal dominant inheritance. (a) Family tree highlighting autosomal dominant cataracts caused by a heterozygous variant in *CHMP4B*, c.481G>C p.(Glu161Gln). Circles represent women, squares men. Filled forms represent affected individuals; unfilled forms unaffected individuals. Crossed forms represent deceased individuals. The question mark in the circle form indicates the phenotype is unknown. Anterior segment photograph of the (b) right and (c) left eye of the proband aged 9 years showing posterior polar cataracts.

Autosomal dominant inheritance is defined by a disease or trait caused by a single heterozygous variant affecting one allele of an autosomal gene. As with autosomal recessive inheritance, these changes can be point mutations, structural changes within a gene, or larger rearrangements/copy number variations which encompass several genes. Autosomal dominant inheritance also does not discriminate between males and females, so both sexes are equally likely to be affected (Figure 2).

Usually, individuals presenting with an autosomal dominant condition will have a family history in keeping with dominant inheritance. In cases where there is no clear family history of the eye condition, this can be due to reduced/non-penetrance, variable expressivity or a *de novo* sporadic pathogenic change in the patient (see below). Each affected individual has a 50% risk for each pregnancy of passing the mutated allele, and therefore the condition, to their child. Consanguinity of parents and family history of the unaffected parent is not relevant in determining inheritance risks in an autosomal dominant disorder.

Among the common pathogenic autosomal dominant genes seen in genetic eye diseases is *RHO*, first described in 1990, mutated in approximately 30% of autosomal dominant RP cases, with the most common variant p.Pro23His.^25–29^ *OPA1* variants account for approximately 65–75% of autosomal dominant optic atrophy, which can be associated with extra-ocular features.^30–32^ *PAX6* variants can cause aniridia, which affects 1:40,000–100,000 births, leading to a variable degree of iris and foveal hypoplasia, nystagmus, cataract, glaucoma and corneal keratopathy.^33,34^

### X-linked recessive disease

X-linked inheritance relates to conditions that manifest as a result of a variant affecting a gene on the X-chromosome. Such conditions primarily affect men through the hemizygous pathogenic mutation, although female carriers can be asymptomatic, mildly symptomatic or display manifest signs of disease, for example, as seen in X-linked RP.^35^ In women, lyonization occurs, commonly referred to as X-inactivation, meaning that one of the X chromosomes is active while the other is inactivated. This is a random process but if more healthy X chromosomes are inactivated in a carrier state, then a woman could be more clinically affected.

**Figure 3.**
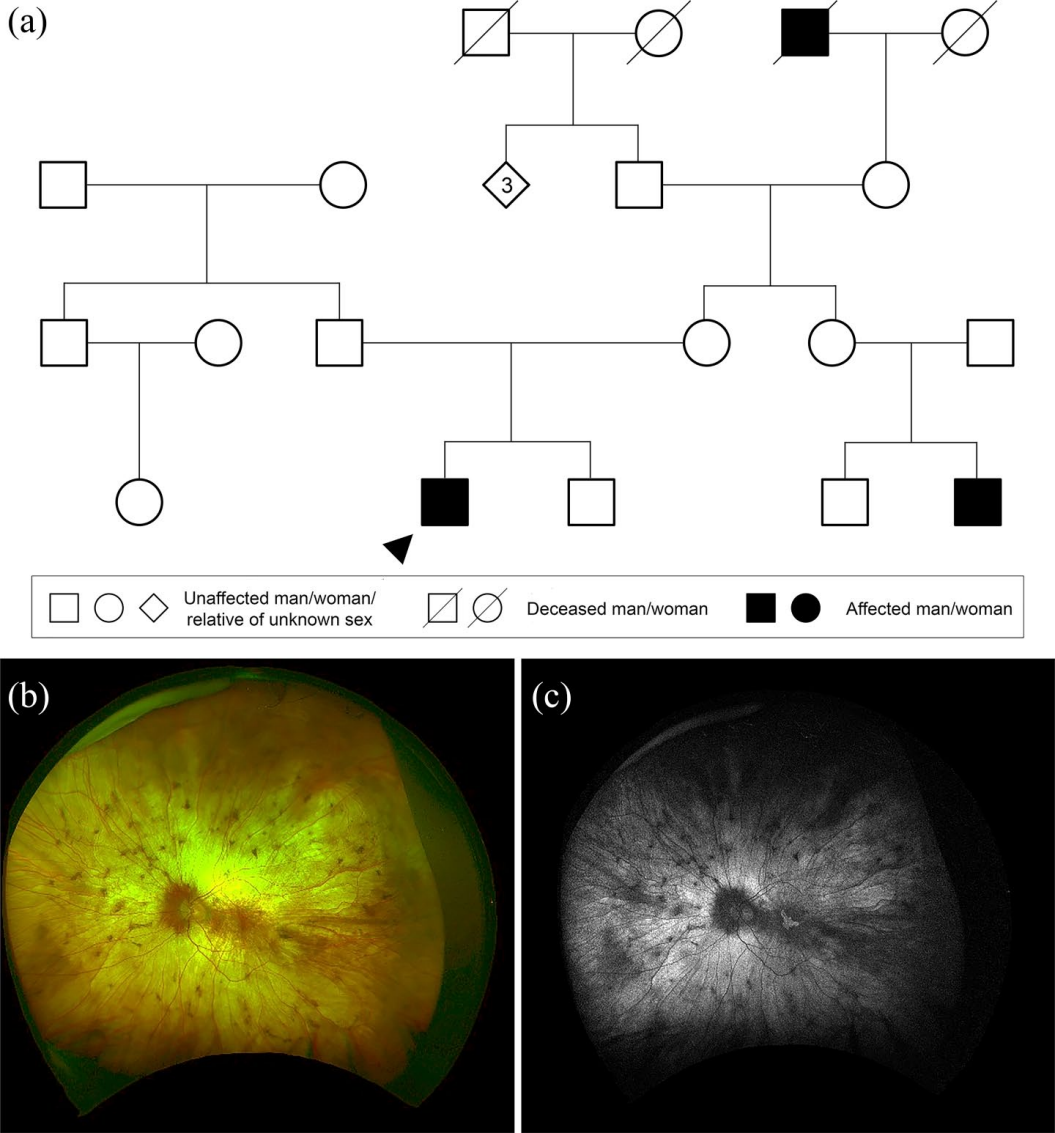
X-linked recessive inheritance. (a) Family tree highlighting X-linked recessive choroideremia caused by a heterozygous variant in *CHM*, c.126C>G p.(Tyr42*). Circles represent women, squares men, diamonds relatives of unknown sex. Filled forms represent affected individuals; unfilled forms unaffected individuals. Crossed forms represent deceased individuals. Numbers in diamond form indicate a number of relatives of unknown sex. (b) Widefield colour fundus photograph of the right eye of the proband aged 31 years showing extensive chorioretinal atrophy with small pigment deposits. (c) Widefield fundus autofluorescence of the right eye showing a small residual island of retina at the macula.

Due to the potential for female asymptomatic carriers of X-linked recessive disease, it is possible for these conditions to appear to ‘skip’ generations on a pedigree through maternal carriers (Figure 3). A male patient with an X-linked recessive disorder will not pass the pathogenic mutation to any of his sons, but all of his daughters will be carriers. It is not possible for man to man transmission of an X-linked recessive condition. A female carrier of an X-linked recessive disorder has a 50% risk of passing the pathogenic mutation to her children, so each of her sons has a 50% risk of being affected and each of her daughters has a 50% risk of being a carrier. It is therefore important to consider the possibility of X-linked disease in sporadic male cases or seemingly dominant pedigrees lacking man to man transmission.

Common X-linked recessive disorders in inherited eye diseases include choroideremia, a rare chorioretinal dystrophy (with a prevalence of one in 50,000–100,000) caused by mutations in *CHM*, characterised by progressive degeneration of the photoreceptors, retinal pigment epithelium (RPE) and choroid.^36^ Variants in *RPGR* are the major cause of X-linked RP, which represents 8% of RP (with a prevalence of one in 3,000–7,000).^37,38^ Lenz microphthalmia syndrome is a X-linked disease characterised by cataracts and microphthalmia, associated with facial dysmorphism, dental and cardiac defects, due to *BCOR* variants.^39^

### X-linked dominant disease

An X-linked dominant condition is also caused by a pathogenic change on the X chromosome. Unlike X-linked recessive inheritance, a female’s healthy X chromosome does not compensate in an X-linked dominant case, so females and males can both be affected. Affected males of X linked dominant diseases are often more severely affected than heterozygous females and a number of such conditions are lethal in males during early life.

A male patient with an X-linked dominant disorder will not pass the pathogenic mutation to any of his sons, but all of his daughters will be affected. It is not possible for male to male transmission of an X-linked dominant condition. A female patient with an X-linked dominant disorder has a 50% risk of passing the pathogenic mutation to her children, regardless of her child’s gender, so each child has a 50% risk of being affected. If a mother has had multiple miscarriages of males, this can be an indicator of an X-linked dominant condition.

X-linked dominant disorders are very rare. One example is incontinentia pigmenti which is caused by pathogenic changes in the *IKBKG* gene. This condition usually only affects females, as it is only in rare cases that males survive.^40^

## Mitochondrial mode of inheritance

Mitochondrial inheritance does not obey the classic rules of Mendelian genetics. The mitochondrial genome comprises a circular ~16.5 kb DNA genome (mtDNA) with 37 genes present in each mitochondrion. The human egg cells, but not sperm cells, contribute mitochondria to the developing embryo; hence, children can only inherit mtDNA mutations from their mother. The severity of the disease is related to the number of mutated mitochondrial DNA in each cell. Mitochondrial disease can affect each generation of a family, with both sexes equally likely to be affected. Of note, fathers do not pass traits associated with changes in mtDNA to their children. Leber hereditary optic neuropathy (LHON)^41^ and maternally inherited diabetes and deafness (MIDD)^42^ are the most common mitochondrial eye diseases, which affect the respiratory chain complex. Kearn Sayre syndrome, characterised by pigmentary retinopathy, ophthalmoplegia and extra-ocular features such as deafness, cerebellar ataxia and heart block, is due to a mtDNA deletion.^43^

## Other inheritance patterns and complexities

Mosaicism occurs when cells within a single individual comprise two or more different genotypes. Somatic mosaicism is a form of mosaicism which occurs following postzygotic mutation.^44^ Germline mosaicism is a form of mosaicism which involves the gamete cells (i.e. sperm or egg). In autosomal dominant cases, germline mosaicism can explain multiple affected siblings from unaffected parents.^45^

A *de novo* mutation is a genetic change that occurs for the first time in an individual which is not present in the parents’ somatic cell DNA. This can be due to germline mosaicism in a parent or the mutation can occur in a fertilised egg during embryogenesis.^45^

Uniparental disomy describes the phenomenon of an individual having two copies of a chromosome, or part of a chromosome, from one parent without the equivalent copy from the other parent.^8–11^ This duplication is usually the result of a meiosis error^46^ and can be seen in one of two forms: uniparental heterodisomy, where the offspring is genotypically identical to a parent at a locus having inherited both alleles carried by the parent, or uniparental isodisomy, where the offspring inherits two copies of a single allele/locus from one parent.

Parental exclusion means the absence of cosegregation between the child and parental genotypes with the most likely scenario being non-paternity or adoption of the affected child. This should be checked for during the genetic consultation and must be taken into account during the genetic analysis to help identify the disease-causing defects.^47^

**Figure 4.**
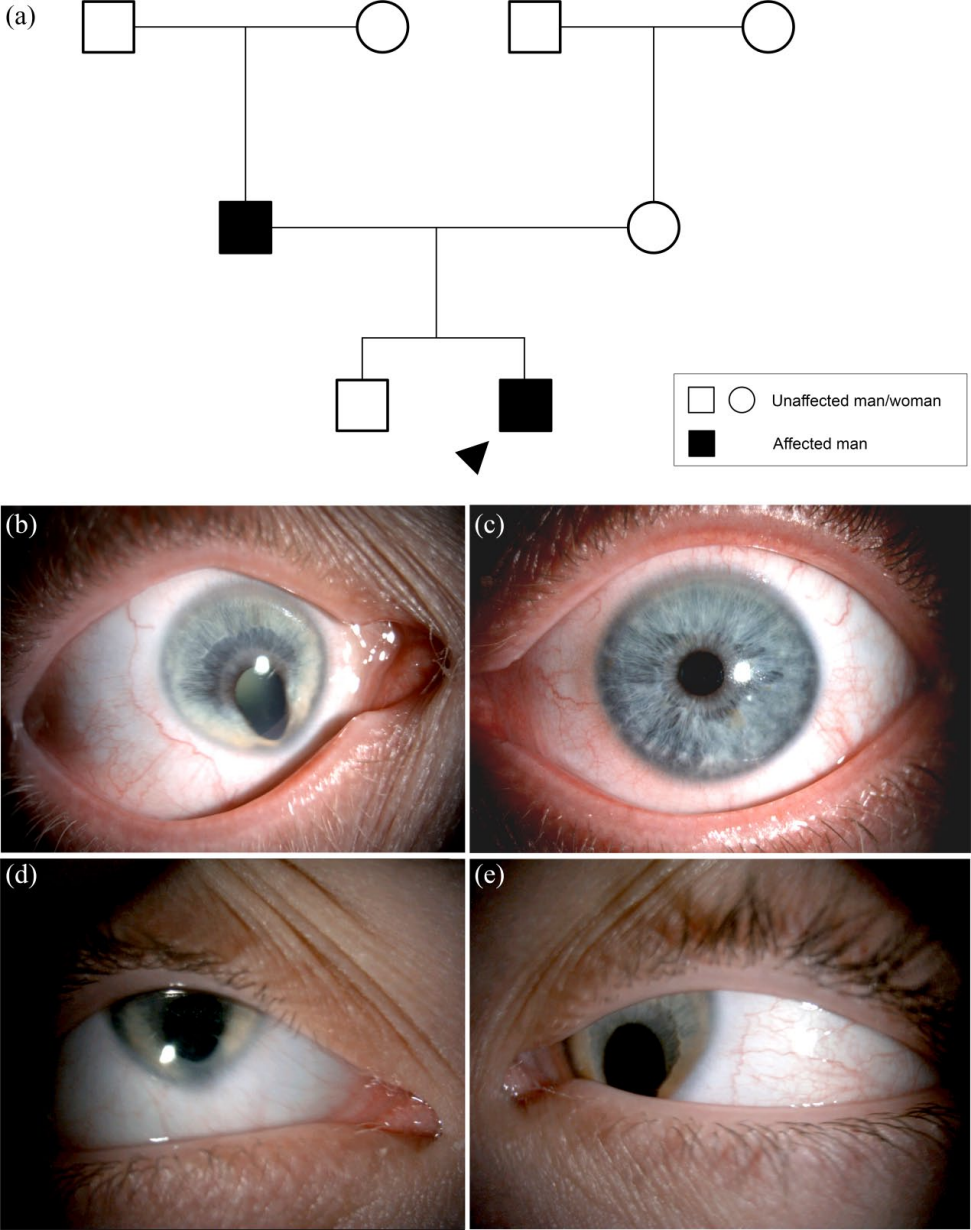
Autosomal dominant inheritance with variable expressivity. (a) Family tree with autosomal dominant non-syndromic microphthalmia with ocular coloboma and variable expressivity between generations. No primary findings following a microphthalmia, anophthalmia and coloboma (MAC) targeted gene panel of 97 genes. Less than 10% of patients with MAC receive a molecular diagnosis. Circles represent women, squares men. Filled forms represent affected individuals; unfilled forms unaffected individuals. Crossed forms represent deceased individuals. (b) Right eye with unilateral right microphthalmia and iris coloboma with microcornea and (c) normal left eye of father. (d) Right and (e) left eye of proband (son) with bilateral microphthalmia, iris coloboma and microcornea.

Variable expressivity means that a pathogenic variant may be associated with varying degrees of phenotypic severity within individuals of the same family, for example, in autosomal dominant non-syndromic microphthalmia and ocular coloboma (Figure 4).

**Figure 5.**
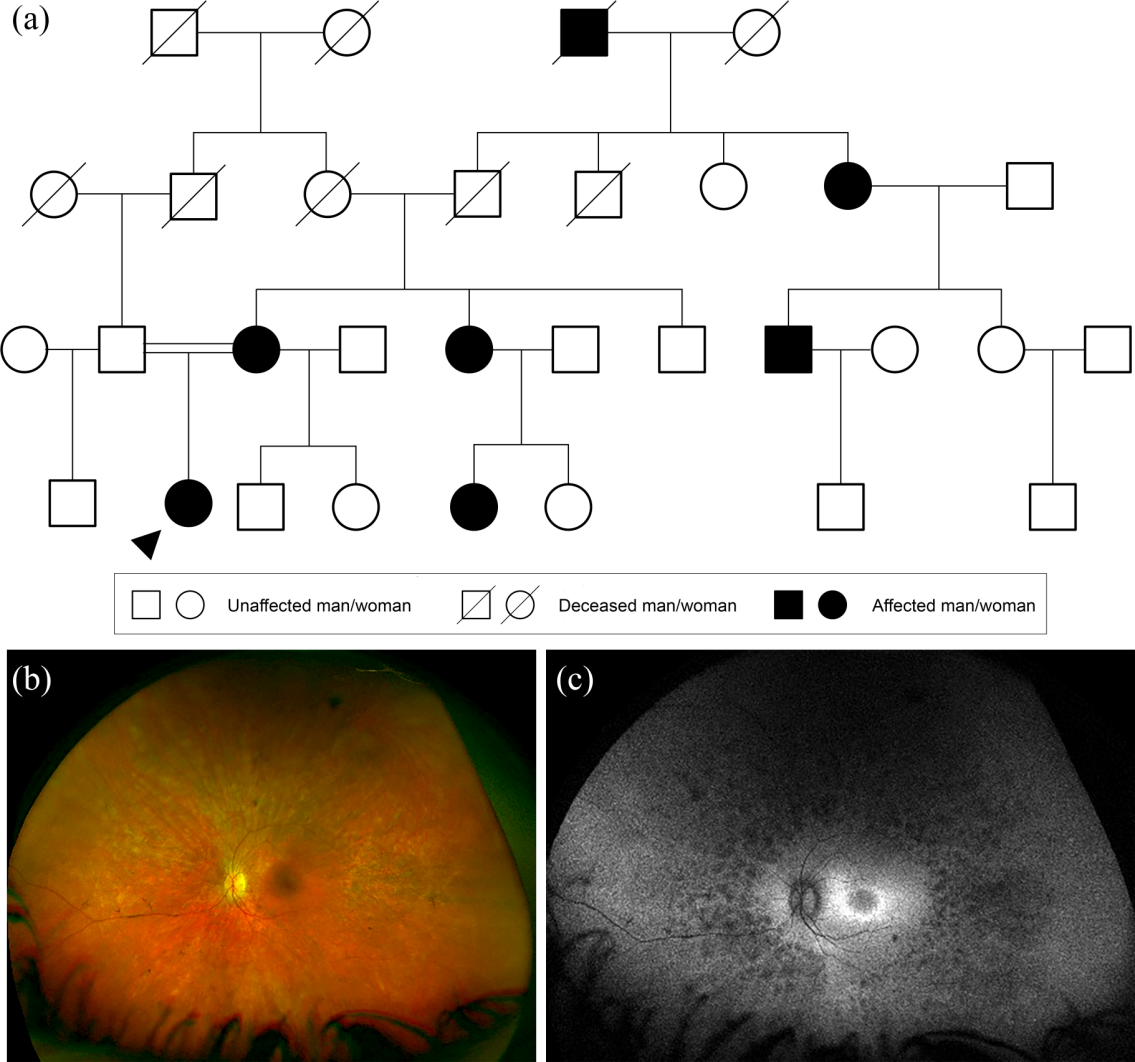
Autosomal dominant with reduced penetrance. (a) Family tree with autosomal dominant retinitis pigmentosa (RP) caused by a heterozygous variant in *PRPF31*, c.547G>T p.(Glu183*). The mother is clinically unaffected but was found to segregate the variant: 50% of gene carriers can be non-penetrant showing no signs of RP. Circles represent women, squares men. Filled forms represent affected individuals; unfilled forms unaffected individuals. Crossed forms represent deceased individuals. Double line represents consanguinity. (b) Widefield colour fundus photograph of the right eye of the proband aged 23 years showing retinal vessel attenuation and scattered mid peripheral bone spicule pigmentation. (c) Widefield fundus autofluorescence of the right eye showing areas of hypoautofluorescence outside the arcades with a perifoveal ring of hyperautofluorescence.

Incomplete or reduced penetrance means that a pathogenic variant does not always result in the patient being affected with the disease.^48^ For example, *PRPF31*, which causes autosomal dominant RP, has been shown to exhibit variable expressivity and reduced penetrance, thus severity of symptoms can vary significantly in affected relatives within the same family with some carriers being totally asymptomatic (Figure 5).^49^

Pseudo-dominant inheritance describes families in which subsequent generations are affected by a recessive disorder and thus appear to follow a dominant inheritance pattern. Pseudo-dominance is more likely to occur in families with consanguinity or in recessive disorders where there may be a high carrier frequency of mutations (perhaps enriched in an isolated community or ethnicity).^50^ Vaclavik and colleagues reported one consanguineous family where the father and two of his three children were affected with enhanced S-cone syndrome, the pedigree appeared autosomal dominant but genetic analyses identified a homozygous variant in *NR2E3* which cosegregated with the phenotype, revealing a pseudo-dominant inheritance.^50^

Disease-onset, severity and prognosis can differ between individuals sharing the same disease-causing variants, known as inter- and intra-familial variability. Environmental factors, genetic modifiers or epigenetic influences may affect the clinical phenotype and explain the difficulty in establishing genotype-phenotype correlations.^51,52^ The field of epigenetics and genetic modifiers is the subject of much research, but clinically relevant discoveries are emerging, for example, in uveal melanoma, patients with a methylated *EFS* promotor have an increased risk of premature death compared with those with an unmethylated *EFS* promotor.^53^ Moreover, some studies report that CpG methylation influences mutation occurrence.^54^ A multiomic approach to studying disease will yield more information on inherited eye disorders than has previously been established, but as yet transcriptomics, metabolomics, proteomics and epigenomics still remain as research-based tests.^55^

## Genetic counselling (part 1) – informed consent and the role prior to genetic testing

**Figure 6.**
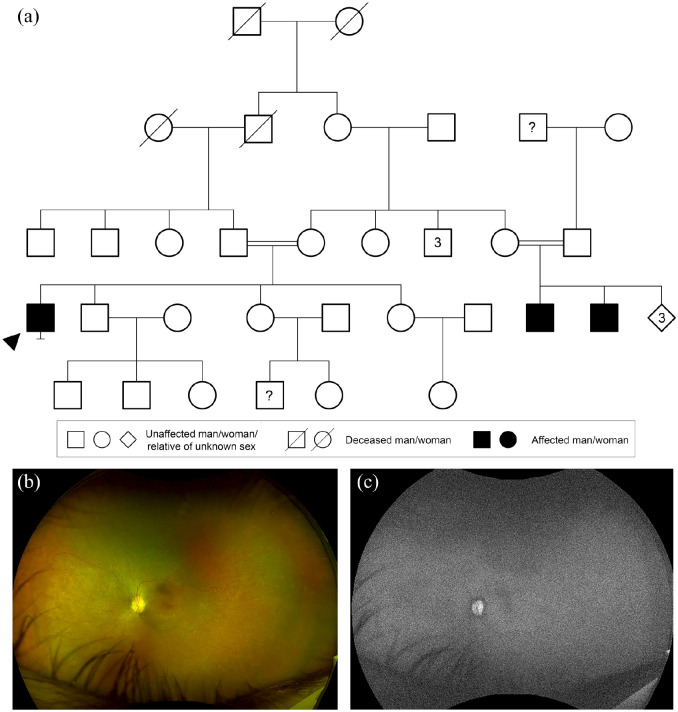
Unclear inheritance – likely either X-linked recessive or autosomal recessive. (a) Consanguineous family tree with retinitis pigmentosa (RP) caused by a homozygous variant in *RPE65*, c.253C>A p.(Arg85Ser). Circles represent women, squares men, diamonds relatives of unknown sex. Filled forms represent affected individuals; unfilled forms unaffected individuals. Crossed forms represent deceased individuals. Numbers in diamond or in square form indicate a number of relatives of unknown sex or of man, respectively. The question mark indicates the phenotype is unknown. Double line represents consanguinity. (b) Widefield colour fundus photograph of the right eye of the proband aged 42 years showing RPE granularity and subtle white dots. (c) Widefield fundus autofluorescence of the right eye showing characteristic lack of autofluorescence.

If a genetic cause is suspected, the clinician should consider the following and discuss this with the patient and their family: (a) Would a molecular result reduce morbidity and mortality through established intervention or access to research? (b) Provide an explanation for their symptoms, should they wish to know? (c) Help to determine the prognosis? (d) Guide family planning?^56^ Genetic counsellors assist clinicians by supporting families with their decision-making on proceeding with genetic testing (Table 1). Depending upon location, genetic counsellors can be registered professionals who must adhere to national guidelines and policies, for example, the Association of Genetic Nurses and Counsellors (UK) and the American College of Medical Genetics and Genomics (USA). They will discuss the concept of genetics, DNA and genetic testing in appropriate language and level of detail for the patients and any relevant family members. The genetic counsellor will discuss a patient’s motivations for undergoing the genetic testing, such as (a) more accurate diagnosis and prognosis, (b) confirmation of inheritance pattern and risks to other family members and (c) potential eligibility for clinical treatment trials, while managing a patient’s expectations. The genetic counsellor will draw a detailed pedigree diagram (genogram), facilitate conversations and counsel patients regarding any ethical concerns (Table 2) and apprehensions they may have. Counselling a patient on inheritance and family risks prior to genetic testing can be challenging, as many Mendelian hereditary eye disorders can be inherited by more than one pattern of inheritance. Accurate discussions about risks to family members and future generations are sometimes only possible after a molecular diagnosis has been obtained (Figure 6). Following the discussion with the genetic counsellor, a patient must make an autonomous decision whether they wish to go ahead with a genetic test (if relevant and available).

**Table 1.** Why pursue genetic testing?

Situation	Aim
Diagnostic testing	To establish a genetic diagnosis for an affected patient with no previously established individual or family result.
Confirmation testing	To confirm a genetic diagnosis of an affected patient; this could be Sanger sequencing for a known genetic result of an affected family member or confirmation in an NHS diagnostic laboratory of a result found previously in research.
Carrier testing	To study the genotype for an unaffected family member of a patient with a known pathogenic change. These tests are most commonly performed in family members of patients with an autosomal dominant gene with reduced penetrance, or for women who are at risk of carrying an X-linked disorder.
Predictive testing	To study the genotype for asymptomatic relatives of affected individuals who are at risk of developing the condition themselves. Current UK guidelines are that asymptomatic children under the age of 16 should not undergo predictive testing for an adult onset disorder.
Familial segregation testing	Might be useful for relatives of patients with an autosomal recessive disease, in order to confirm that two pathogenic mutations are in *trans*.

**Table 2.** Ethics issues associated with genetic screening.

	Ethics issues
Presymptomatic testing in children	A parent or guardian of a clinically unaffected child known to be at risk of a genetic eye disorder (e.g. a younger sibling of an affected patient) may wish to find out whether their child will develop the condition. This raises ethical concerns, especially for conditions where there is no treatment or management available and where the usual onset of symptoms is in adulthood. A parent making this decision on behalf of a child is removing the autonomy of the child to make its own decision. Genetic counsellor guidelines state that presymptomatic testing in children will only be offered in cases where there is a clinical need (e.g. treatment or prevention availability).
Presymptomatic testing in adults	If a patient is currently asymptomatic or only very mildly affected, there is the potential for a negative psychological impact of a positive result. This decision needs to be balanced against the anxiety of inheriting a known diagnosis in a family and possible treatments or lifestyle adjustments.
Choice and expectations	It is important that genetic testing is presented to a patient as a choice, rather than mandatory, just like any other procedure for which the risks and benefits must be discussed to arrive at informed consent. The clinician and genetic counsellor must make sure that a patient’s expectations regarding genetic testing and results are appropriately managed.
Informed consent (capacity and phrasing)	The informed consent process should be a two-way conversation between a genetic counsellor and a patient. This can lead to ethical challenges regarding patients of various educational backgrounds understanding the relevant genetic principles.^a^ Some patients, such as those with severe learning difficulties, might not have the ability to provide informed consent for genetic testing which leads to ethical concerns about whether it is appropriate for family or medical professional to make a decision on their behalf in cases where there is no clinical benefit or treatment option available for the patient.
Identity	An ethical issue that arises, particularly during family planning discussions, is that a patient might consider their diagnosis to form part of their identity. Therefore, discussions about ‘risks’ and reproductive options might have implications on a patient’s feeling of self-worth. Using appropriate language and a patient-led approach can help to minimise these problems, for example, a genetic counsellor may choose to talk about ‘chances’ of a patient’s child inheriting a condition rather than ‘risks’.
Blame/responsibility	A positive genetic result may lead carrier parents to feel a burden of responsibility for their child’s diagnosis. This is something that should be considered and discussed before genetic testing is offered to a family.
Family implications	Having a genetic diagnosis can lead to a patient learning of risks of other relatives developing the same disorder. Feedback of results needs to be handled carefully by a genetic counsellor and support should be offered to relevant family members. Family members can sometimes put pressure on an affected relative to pursue genetic testing so that they can learn of their individual risks. It is important that the affected patient is making an autonomous decision about genetic testing and is not merely responding to pressure from family members.
Unexpected paternity or family relationships	When genetic screening occurs in multiple family members, this can reveal a lack of paternity or other unexpected familial relationships of which the patient and other family members may not be aware.^b^
Social and legal implications	If a patient obtains a genetic diagnosis, there can be social and legal implications which should be considered prior to a decision being made about testing. These implications can be worrying for patients and include concerns about health/life insurance, driving and employment.
Data ownership, storage and privacy	Significant ethical concerns, which are more relevant with the increase of whole exome/genome sequencing, relate to the data produced from genetic testing.^b^ Key questions to be considered by patients are: What data are stored? How and where is it stored? What methods are put in place to keep the data secure? Who has access to the data and how can it be used? Concerns about data use and misuse are a common concern of patients which genetic counsellors need to discuss in detail during the informed consent procedure.
Incidental findings	Genetic testing often gives rise to the possibility of unexpected incidental findings.^b^ These could be syndromic features linked to the clinical diagnosis, or linked to a separate diagnosis. This can be very distressing for a patient and may require referrals to other specialist clinicians for screening, management or treatment.

a Tomlinson AN, Skinner D, Perry DL, *et al.* ‘Not tied up neatly with a bow’: professionals’ challenging cases in informed consent for genomic sequencing. *J Genet Couns* 2016; 25: 62–72.

b Ormond KE, Wheeler MT, Hudgins L, *et al.* Challenges in the clinical application of whole-genome sequencing. *The Lancet* 2010; 375: 1749–1751.

## Clinical genetic testing

Clinical genetic testing encompasses next generation sequencing (NGS), and cytogenetic testing. Ultimately, retrieving a molecular diagnosis allows both patients and health care professionals to have a better understanding of the disease, establish genotype–phenotype correlations, which will inform clinical management and help determine a prognosis. Each country has its own framework for genetic testing. In the United Kingdom, all clinical-grade genetic testing must be performed within the approval of the United Kingdom Accreditation Service (UKAS). This is the only national accreditation body that is recognised by the government, which inspects and accredits clinical laboratories against internationally agreed standards. Molecular results are interpreted, verified and reported by Health and Care Professional (HPCP) registered clinical scientists. As of October 2018, all genetic testing within NHS England was reconfigured with the aim to provide a single national testing network, with genetic tests undertaken by one of seven genomic laboratory hubs.^57,58^ To aid test selection, a National Genomic Test Directory for rare and inherited diseases has been curated detailing which test is available for each clinical indication (https://www.england.nhs.uk/publication/national-genomic-test-directories/). The Genomics England Panel app lists the genes included in the targeted gene panels.^59^ All genes classified as ‘Green’ in Panel app have undergone review by a panel of experts and deemed to have enough evidence to be included on a diagnostic gene panel for a disease. In Europe, as in the United Kingdom, clinical genetic testing must be approved by the European co-operation for Accreditation (http://www.european-accreditation.org/) with some guidelines and advice provided by the European Society of Human Genetics (ESHG, https://www.eshg.org/index.php?id=home) and the European Reference Network for Rare Eye Disease (ERN-RED, https://www.ern-eye.eu/).^60^In the United States, Clinical laboratory Improvement Amendments (CLIA) provides validated laboratory procedures, while Clinical and Laboratory Standards Institute (CLSI) standardises tests.^60,61^

For most ophthalmology conditions, sequencing of either a single gene, for example, *PAX6* for aniridia in adults, or targeted gene panels, for example, genetically heterogeneous conditions such as retinal dystrophies, is typically recommended as the primary route of molecular analysis. For syndromic conditions alternative genetic testing methods such as genome wide copy number variant (CNV) analysis by microarray may be more suitable.

## NGS

The primary approach for the investigation of genetically heterogeneous eye disorders is through NGS. This uses massively parallel sequencing technology which enables the parallel sequencing of multiple targets from multiple samples (known as multiplexing),^62^ providing a cost-effective method for genetic testing. One of the most frequently used platforms for NGS are those developed by Illumina; this is characterised by the method of DNA fragment amplification on a flow cell, known as ‘bridge amplification’.^62^ In brief, NGS permits the sequencing of multiple short DNA fragments (averaging 150 bp in length), these fragments of DNA are then bioinformatically aligned to a reference genome. Variation between the aligned sequenced DNA and the reference genome is identified or ‘called’, filtered for quality and annotated [with data from external databases such as population frequency information from the Genome Aggregation Database (gnomAD; https://gnomad.broadinstitute.org/)],^63^ before being analysed by a clinical scientist (see below).

**Figure 7.**
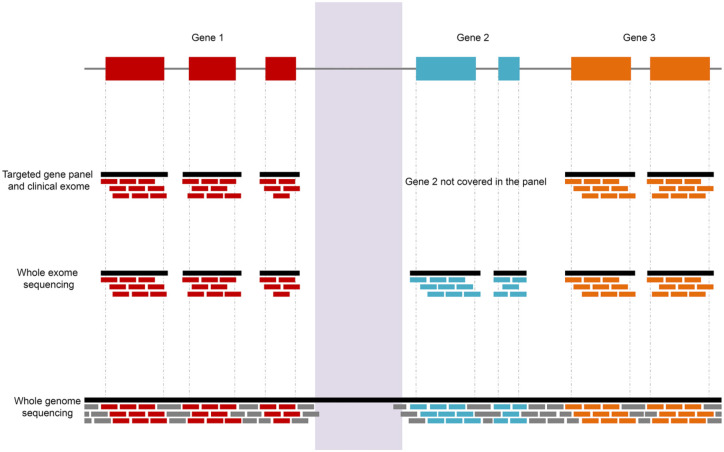
Schematic highlighting the different coverage of NGS including targeted gene panels, whole exome and genome sequencing. Gene 2 is not included in the targeted gene panel. The pale purple region corresponds to a region difficult to sequence, not covered by NGS approaches, including whole genome sequencing. Each horizontal bar, called a ‘read’, is aligned to the reference genome and this alignment allows the identification of variants and copy number variations (CNVs).

The use of NGS is now widely used, but there are several different testing options available, which are defined by the capture method used to select and enrich the regions of interest in the DNA, prior to sequencing; these include (Figure 7) the following:

1. Targeted gene panels or clinical exome;
2. Whole exome sequencing (WES);
3. Whole genome sequencing (WGS).

### Targeted gene panels or clinical exome

Targeted sequencing utilises a DNA capture and enrichment chemistry that specifically selects regions of interest or post sequencing ‘virtual gene panel’ targeting to focus analysis on a smaller subset of genes.^64–66^ These panels are custom designed to target exons and flanking intronic regions of genes previously reported to be associated with genetic eye diseases. In genetic diagnosis, targeted next-generation sequencing is also called a clinical exome. For example, a targeted gene panel, named the Oculome, was designed to screen 429 known eye-related disease causing genes following clinical exome capture and sequencing.^64^ Developed by a collaboration of experts with the aim of maximising the chances of detecting pathogenic mutations with a single genetic test and chemistry, the Oculome is divided into subsets relating to the clinical presentation, for example, if a patient has congenital cataract, then the cataract and lens abnormalities subpanel will be selected. A study of 277 patients who had congenital eye disorders screened with the Oculome provided a definitive diagnosis in 68 individuals (25% diagnostic rate).^64^ CNV analysis was also performed by analysing and comparing the read depth of exons, but the identification of breakpoints was not possible unless within the covered regions: any CNVs of potential clinical interest require microarray analysis for validation.^64^ A similar inherited eye disease panel was developed, covering exons, flanking introns and 5′- or 3′-untranslated regions with specific deep intronic regions of 214 associated genes; of the 192 patients tested, a disease-causing variant was found in 51% of cases.^65^ Genes associated with IRD were reported in the Retinal Information Network database (RetNet; https://sph.uth.edu/retnet/home.htm).

The use of targeted gene panels is a cost effective way to maximise coverage of relevant genomic regions and genes. The benefits including (a) sequencing targeted regions with a greater read depth, meaning that lower frequency variants or mosaicism are more likely to be detected; (b) generating smaller data files requiring less computational and bioinformatic processing and less data storage; and (c) variants identified are targeted and thus more likely to be clinically relevant.^67^ However, the biggest limitation of targeted gene panels is the frequency of their updates: if a novel gene or variant has been associated with a particular genetic eye disease, it will not be sequenced until added, but this requires the redesigning, re-ordering and re-validation of the panel before clinical use.^68^ The most effective way to update gene panels of newly discovered genes is by using whole exome or whole genome capture with virtual gene panel testing (see below). The validation of novel candidate genes associated with inherited eye diseases relies upon the identification of disease-causing variants in candidate genes in multiple unrelated affected individuals. Candidate genes can be added to gene panels for identification of additional cases, which can help to diagnose rare conditions. However, this approach is less commonly used to identify novel gene defects compared with whole exome or genome sequencing.

Useful online resources that will assist in understanding the mode of inheritance, selection of disease genes and the appropriate management include Genetics Home Reference (https://ghr.nlm.nih.gov/); Clinical Genome Resource (https://clinicalgenome.org/); and Gene Vision (www.gene.vision).

### WES

WES is defined by the selection, enrichment and sequencing of exons and flanking intronic regions of known protein coding genes within the human genome, and permits the use of a single capture kit. Although the exome makes up a very small proportion of the genome (~1.5%), it is estimated that over 85% of disease causing variants are within protein coding regions.^69,70^ Interestingly, all exons are covered which allow the identification of novel gene defect(s) associated with the disease.^71–75^ The exon coverage data can be used to analyse CNV, by interrogation of read depth, and may help to identify a duplication or deletion in a gene previously reported to be associated with genetic eye diseases.^49^ This has limitations in that the average read depth generated by standard WES protocols may not be sufficient to characterise many loss or gains. In addition, inversions, translocations, complex and noncoding rearrangements are intractable and breakpoints are usually not covered making validation more difficult. Despite only accounting for around 1.5% of the genome, the data generated are large. Access to a high-performance computing (HPC) cluster and significant data storage can alleviate bioinformatic challenges and bottlenecks in the processing, analysis, storage and interpretation of the data.

### WGS

WGS far exceeds the targeted coverage offered by the enrichment methods described above. WGS methods omit polymerase chain reaction (PCR) amplification of targeted and exome capture, thereby enabling coverage of PCR-intractable genomic regions including GC rich exons.^74,76–79^ The complete coverage of the genome, over 3 billion nucleotides and 20,000 genes, afforded by WGS allows the identification of previously noncovered variants such as CNVs, structural variations, intergenic and deep intronic variants.^80–85^ Recently, the 100,000 Genome Project was undertaken by Genomics England supported by NHS funding and infrastructure in England.^86–88^ The objective of this project was to kick-start genomic medicine in the United Kingdom by generating genome data from 100,000 individuals across rare disease and cancer to improve clinical diagnostics for patients.^89^ Of note, the cost of WGS and its analyses and interpretation is far higher than for an exome.^90^ But as with WES or targeted gene panel testing, exons and flanking intronic regions of genes can be screened first to identify any disease-causing variants in these regions^80^ and thus, these methods are currently favoured by clinical genetics service laboratories and researchers as the first-line approach, reserving the rest of the genome for only cases unsolved by targeted screening.

## Variant interpretation and reporting

### Analysis of NGS data

Following NGS, the raw sequencing data are processed through bioinformatic pipelines in order to generate a human readable dataset of variants. There are numerous quality checks and controls in place to inform the scientist on the quality of the sequencing, alignment and variant calling. The coverage indicates how well and what portion of a gene has been sequenced at a specified read depth. For example, if the coverage of a gene is reported to be 95% at 20×, this means that 95% of the gene has more than 20 sequencing reads mapping to it. This control ensures that all regions of interest are sequenced to an adequate depth.

### Bioinformatic pipelines

In brief, the bioinformatic pipelines have several steps characterised by the following:

1. Alignment: The mapping of the short sequencing reads to a reference genome achieved *via* tools such as BWA-mem (http://bio-bwa.sourceforge.net/). This produces a file known as a binary alignment file (BAM) which can be viewed using tools such as integrative genomics viewer (IGV; https://igv.org/), which is useful when assessing variants.
2. Variant calling: The process of identifying variation between the sequenced DNA and the reference DNA, *via* tools such as Genome Analysis Toolkit (GATK, https://software.broadinstitute.org/gatk/). This produces a variant call file (VCF), which is a text file listing all variants identified, their genomic position, the nucleotide change observed, and additional information relating to the quality/confidence of the variant call.
3. Variant annotation: The process of collating and annotating each variant in the VCF with evidence from multisources required to aid interpretation^63,91–98^ (Table 3).
4. Optional: Virtual panels and variant filtering to aid variant interpretation and reduce the number of variants requiring time intensive variant interpretation. Unlike targeted panels which specifically screen targeted regions, virtual panels are a bioinformatic filter applied post sequencing and variant calling, to filter and select variants within a defined list of genes or regions of interest. Virtual panels correspond to a list of regions, frequently supplied in the form of a BED file (a tab delimited file detailing the chromosome, start and stop position in a standardised format) which aids analysis by restricting variants to the region of interest and reduce the risk of incidental findings. Virtual panels are often applied to large targeted panels, whole exome or genome sequencing,^64,71,72,80,99^ and as this is a bioinformatics approach, the panel can be easily edited to include or exclude genomic locations, recently published genes,^71,72^ candidate genes, without the need to re-run sequencing. Thus, variant lists can be filtered to remove common polymorphisms and focus on rare variants and those affecting highly conserved residues/domains among species.^100^ A lot of effort has been spent on designing and curating the virtual panels for clinical genetic analysis and has resulted in collaborative projects such as the Genomics England Panel app project (https://panelapp.genomicsengland.co.uk/), which aims to curate lists of clinical relevant genes associated with different phenotypes, lists vetted and tiered by experts within the scientific and clinical community.^59^

**Table 3.** Variant annotation uses multiple sources.

Genome aggregation database (gnomAD)^63^	https://gnomad.broadinstitute.org/	Population database providing allele frequency for variants across different ethnic backgrounds. For each variant, each ethnicity and each sex, number of homozygotes and allele frequency is calculated. Rare variant frequency is below 1%.
UCSC Genome browser	https://genome.ucsc.edu/cgi-bin/hgTracks?db=hg38&position=lastDbPos	Amino acid or nucleotide conservation, across 100 species. Highly conserved amino acid/nucleotide is more likely important in protein function, splicing, mRNA integrity and so on.
ClinVar	https://www.ncbi.nlm.nih.gov/clinvar/	Disease association: variants reported in the literature.
Human Gene Mutation database (HGMD)	http://www.hgmd.cf.ac.uk/ac/index.php	Disease association: variants reported in the literature.
Sorting Intolerant From Tolerant (SIFT)^101^	http://sift.jcvi.org/	*In silico* prediction algorithms (non–splice site variants), based on disease association, sequence homology, amino acid conservation across species and conserved region, amino acid physicochemical characteristics, three dimensional structure. Variants can be predicted as tolerated or deleterious.
Polymorphism Phenotyping v2 (PolyPhen2)^102^	http://genetics.bwh.harvard.edu/pph2/	*In silico* prediction algorithms (non–splice site variants), based on disease association, sequence homology, amino acid conservation across species and conserved region, amino acid physicochemical characteristics, three dimensional structure. Variants can be predicted as benign, possibly or probably disease-causing.
Align GVGD^103,104^	http://agvgd.hci.utah.edu/agvgd_input.php	*In silico* prediction algorithms (non–splice site variants), based on disease association, sequence homology, amino acid conservation across species and conserved region, amino acid physicochemical characteristics, three dimensional structure. Variants are classified if they most likely interfere with the function of the protein.
MaxEntScan^105,106^	5′: http://hollywood.mit.edu/burgelab/maxent/Xmaxentscan_scoreseq.html 3′: http://hollywood.mit.edu/burgelab/maxent/Xmaxentscan_scoreseq_acc.html	*In silico* prediction algorithms (splice site variants), based on consensus motif and maximum entropy principal. Higher score indicates a higher probability that the sequence being a splice site.
Splice Site Prediction by Neural Network (NNSPLICE)^106,107^	http://www.fruitfly.org/seq_tools/splice.html	*In silico* prediction algorithms (splice site variants), based on generalised Hidden Markov Model (GHMM). Higher score indicates a potential splice site.
Human Splicing Finder (HSF)^106,108^	http://www.umd.be/HSF/#	*In silico* prediction algorithms (splice site variants), based on nucleotide conservation. Higher score indicates a potential splice site.

Collection of this evidence is frequently undertaken by tools such as Alamut (https://www.interactive-biosoftware.com/alamut-batch/) or variant effect predictor (VEP, https://www.ensembl.org/info/docs/tools/vep/index.html).

GHMM, generalised Hidden Markov Model; HGMD, Human Gene Mutation database; HSF, Human Splicing Finder; NNSPLICE, Splice Site Prediction by Neural Network; SIFT, Sorting Intolerant From Tolerant.

### Variant interpretation

Following bioinformatic processing, each variant identified is analysed to establish its possible association with the disease phenotype. Clear and concise phenotype information must be supplied with any genetics referral. All variants are analysed by a clinical scientist and independently checked by a second clinical scientist, using the information supplied through the annotation step of the bioinformatic pipeline (see above) and additional investigation of databases and publications. In general, data can be filtered following the mode of inheritance, the type of variants, the frequency in large-scale exome and genome sequencing datasets, the pathogenicity prediction, amino acid or nucleotide conservation across species, relevant tissue expression, protein localisation and prior related publications and mutation databases. The allele frequency filter should be applied considering the rarity of disease, inheritance of the gene/mutation, and thus dominant disease analysis may differ from recessive disease/gene filtering strategies. The variant rarity is the most important feature to help to identify the disease-causing variant. For example, if the frequency of an autosomal dominant disease is one in 10,000, then the frequency of variant must be less than one in 10,000. Of note, the frequency of known mild mutations or reduced penetrant variants might be higher, as the *ABCA4* variant, c.5882G>A p.(Gly1961Glu) is highly frequent in Somalians (~10%).^19^ A virtual panel can also be applied to focus the analyses on genes previously reported to be associated with the disease, recently published genes and candidate genes, which reduces the number of putative disease-causing variants. The number of variants is highly dependent on the applied methods and varies between thousands to millions, which highlights the importance of variant filtering to reduce the data to a manageable size.

Variant pathogenicity interpretation has been standardised and classified using the five-class system (Class 5 Pathogenic; 4 Likely pathogenic; 3 Variant of unknown clinical significance; 2 Likely benign; and 1 Benign) for small nucleotide variants and small insertions/deletions (indels)^109^ and this classification was also adapted for CNVs in single genes.^110,111^

When no disease-causing variants are identified in known genes associated with inherited eye disorders, cases must be re-analysed for novel genes. High impact variants outside the panel can be uploaded to various data sharing platforms including GeneMatcher^112^ with the aim of identifying supporting data for causality of novel gene variants in similarly affected individuals for a large collaborative network of researchers. Moreover, syndromic genes or related gene panels may be considered, for example, if a patient has microphthalmia and anterior segment dysgenesis (ASD), if the MAC panel has no primary findings, the patient could also be screened with the ASD panel.^113^

## Cytogenetic testing

Cytogenetic testing can be used to verify NGS findings or to detect chromosomal abnormalities or CNVs.^114^ Cytogenetic testing can include karyotyping, microarray-based comparative genomic hybridisation (array-CGH), fluorescent *in situ* hybridisation (FISH, used to detect and localise the presence or absence of specific DNA sequences on a chromosome, for example, in ocular lymphoma or melanoma^115,116^) and qualitative fluorescent polymerase chain reactions (QF-PCR, used to amplify specific regions of DNA to quantify and confirm the copy number in that specific region, previously reported with NGS approaches, and can identify common aneuploidies).^117^

Array-CGH is a more detailed and sensitive technique that looks for CNVs.^114,118^ Array-CGH can detect abnormalities between 100,000 base pairs (100 kb) to more than 5,000,000 bp (5 Mb) by comparing with a normal reference genome.^114^ Studies have shown that array-CGH has shown to have higher detection rates for patients with syndromic-related ocular diseases and is often the initial genetic test performed in such individuals. Array-CGH is commonly used in children presenting with aniridia to detect a deletion involving the *WT1* and *PAX6* genes, if negative then Wilms tumour, aniridia, genitourinary anomalies and mental retardation (WAGR) syndrome can be ruled out. Then single gene PCR-based sequencing of *PAX6* is undertaken to identify pathogenic variants causing isolated aniridia.^34,119^ Array-CGH has been found to be better at detecting CNVs in comparison with FISH and QF-PCR.^120,121^ It may also identify incidental findings for unrelated genetic conditions; however, it is not able to detect balanced rearrangements or mosaicism.

Karyotyping is one of the most conventional ways of testing for chromosomal abnormalities.^122,123^ It can only detect large anomalies (minimum size: 5–10 Mb) like deletions, inversions or duplications.^124,125^ It is commonly used in prenatal testing for the detection of Down’s syndrome which is related to many ocular conditions including strabismus, refractive error, nystagmus, eyelid malposition, corneal ectasias, iris nodules (Brushfield spots), presenile cataracts, glaucoma and retinovascular anomalies.^126–128^

## Epigenetic testing

This is not yet considered an accredited clinical test in the United Kingdom for ophthalmic Mendelian disease, but can be conducted in research studies. Several approaches, such as methylation sensitive micro-arrays or bisulfite sequencing, exist to study the methylation profile in specific regions or across the whole genome.^129^ Methylation is an important epigenomic biomarker that exerts a reversible chemical modification, most commonly at cytosine residue in CpG dinucleotide sequences in DNA. Methylation sensitive microarrays use methylation-sensitive restriction enzymes to identify methylated fragments.^129^ This approach needs a large quantity of DNA (1–10 µg) to provide methylation profile at the whole genome level and to cover several hundred CpG islands. Bisulfite sequencing converts methylated cytosine into uracil in CpG sites.^129^ Specific fluorescent-labelled primers are designed to hybridise the unmethylated or methylated allele and the methylation profile is visualised by microarray hybridisation or by whole genome amplification.

## Confirmation of variants

Class 4 or 5 variants identified by NGS may be confirmed by Sanger sequencing, although more frequently this step is being omitted when scientists are confident in the quality reports from the NGS pipeline. Sanger sequencing is a targeted sequencing method able to sequence approximately 500 bases at a time, after an amplification step by PCR of the region of interest. This approach is needed to confirm the variant and to perform segregation studies when DNA from affected and unaffected family members is available. Any potential CNVs of clinical interest detected *via* NGS should be confirmed by a clinically validated method, such as multiplex ligation-dependent probe amplification (MLPA) to detect copy loss or gain of single exons of a gene, array-CGH (microarrays) or QF-PCR, and reported as an outcome of those tests.

## What does a genetic report look like and how is it interpreted?

**Figure 8.**
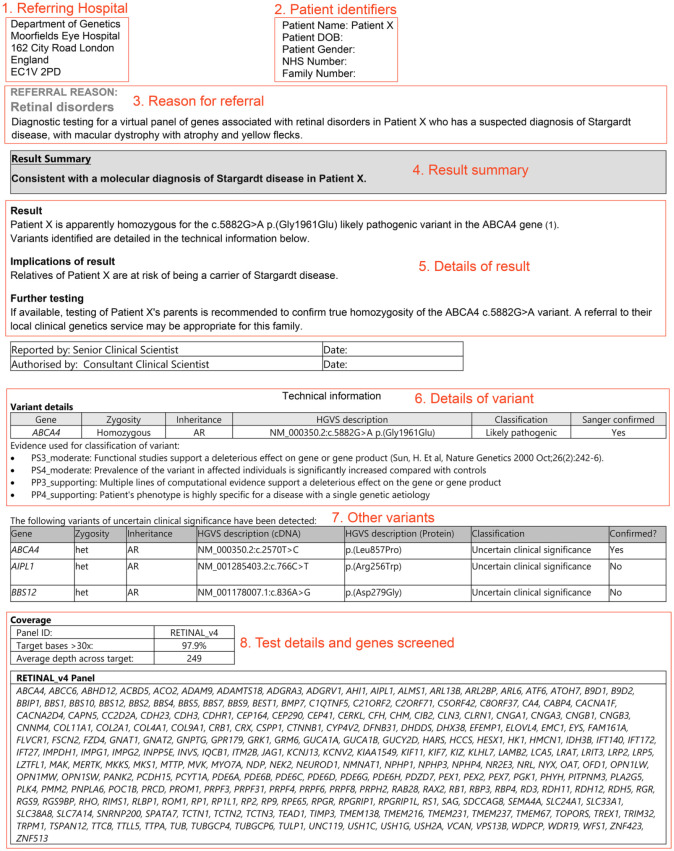
Example of a clinical report. Clinical reports clearly display the overall result, with further information of the evidence used to reach the conclusion. The name of the test laboratory will be at the top of the report but this has been removed in this example. The clinical report must indicate the referring clinician and their address (1); patient details (2); test/referral reason (3) in this case a patient presenting a suspected diagnosis of Stargardt disease; a result summary (4); further details (5) on the disease-causing variant(s), the state of zygosity, the variant classification, the implication of results and further testing which must be clearly explained at the future appointment; variant(s) details (6) which list the disease-causing variants and associated gene, its mode of inheritance, the gene reference, the variant classification and its evidences with publication references; other variants of unknown pathogenic significance (7) identified with the test; and test details (8) listing the genes screened and the depth and coverage reached.

Clinical genetic reports are formal documents communicating analytical results of a sample to the referring clinician and must conform to the Association for Clinical Genomic Science (ACGS) guidelines for best practice.^130^ All clinical reports must be approved and authorised by a senior clinical scientist, prior to being sent to the referring clinician. Clinical reports clearly and concisely display the overall result, with further evidence used to reach the conclusion detailed below (Figure 8). It should be noted that only confirmed variants associated with the patient’s phenotype would be reported within the main body of the report (Box 2). Analysed variants classified as class 3 ‘Variants of uncertain clinical significance’ will frequently be listed in the appendix of the report.

Box 2. **Guide to interpreting variant nomenclature**

The identified variants must be reported using standardised nomenclature set out by the human genome variation society (HGVS, http://varnomen.hgvs.org/).^133^ Here is an example of how to interpret a variant, for example, *ABCA4* NM_000350.2 c.5882G>A, p.(Gly1961Glu), the mutation reported in Figure 8:

1. The gene name is written in *italics* and capital letters (*ABCA4*)
2. This is associated with a transcript reference sequence, starting with a NM number (NM_000350.2) which helps to relocate the variant if required.
3. The nucleotide change is a indicated by the prefix ‘c’. for complementary DNA (cDNA) reference sequence, this is followed by the position of the variant counted from the first nucleotide A of the ATG start site (c.5882)
4. At this position c.5882 the nucleotide change is written, in this case a G is changed to an A (G>A).
5. Next the effect on the protein is given denoted as ‘p’. for protein reference sequence. The original amino acid is given first and its position (counted from the start codon AUG or methionine, Met) followed by the result due to the nucleotide change. For example, p.(Gly1961Glu) denotes that in the protein sequence at amino acid position 1961 a glycine (Gly) was changed to a glutamic acid (Glu); this is a missense mutation.

Further common examples, as nonsense, in splice site, deletion, duplication, insertion and deletion/insertion, are detailed in the table below.

Common examples of variant nomenclature

**Table:** 

Type of mutation	DNA level (c.)	Protein level (p.)
Substitution (missense)	c.612A>G cDNA; position substituted; reference nucleotide; >; mutated nucleotide *At position 612, the A nucleotide is changed to a G*	p.(Ser45Thr) Protein; reference amino acid; position substituted; mutated amino acid ***Amino acid residue at position 45, a serine (Ser) is changed to a threonine (Thr)***
Substitution (nonsense)	c.1257C>A **cDNA; position substituted; reference nucleotide; >**; **mutated nucleotide** *At position 1257, the C nucleotide is changed to an A*	(Tyr419*) **Protein; reference amino acid; position substituted; stop codon** Amino acid residue at position 419, a tyrosine (Tyr) is changed to a stop codon (* or Ter)
Splice site	c.6729+2A>G **cDNA; position substituted; reference nucleotide; >**; **mutated nucleotide** *At position 6729+2 (2 bp upstream of the exon within the intron), the A nucleotide is changed to a G*	*Splice variants affect the splicing of the transcript resulting in retention of intronic DNA or entire exons being spliced out, resulting in abnormal protein which is not usually denoted*
Deletion	c.186delGA **cDNA; position deleted; deleted nucleotide/s** *At position 186, the two consecutive nucleotides GA are deleted*	p.(Asn62Argfs*31) **Protein; amino acid(s)+position(s) deleted; del** Asparagine (Asn) at amino acid position 62 is changed to arginine (Arg). The deletion of two nucleotides leads to a frameshift (fs) where a stop codon (*) appears 31 amino acid residues after position 62
Duplication	c.754_756dup **cDNA; position(s) duplicated; dup** *The nucleotides between position 754 and 756 (included) are duplicated*	p. Met252dup **Protein; amino acid(s) + position(s) duplicated; dup** *The methionine (Met) at position 252 is duplicated*
Insertion	c.125_126insAACT **cDNA; positions flanking; ins; inserted sequence** *The nucleotides AACT are inserted between the nucleotides 125 and 126*	Arg159_Pro160insLeu **Protein; amino acids + positions flanking; ins; inserted sequence** ***A leucine (Leu) is inserted between the arginine at position 159 and the following amino acid proline (Pro) at position 160***
Deletion/Insertion	**c.345_366delinsGCCT** **cDNA; position(s) deleted; delins; inserted sequence** *The nucleotides between the position 345 and 366 (included) are deleted and the nucleotide GCCT are inserted*	**Arg159_Pro168delinsLeu** **Protein; amino acid(s)+position(s) deleted; delins; inserted sequence** ***The amino acids between position 159 and 168 (included) are deleted and a leucine (Leu) is inserted.***

a If the variant is upstream of the exon, the variant must be identified as the last nucleotide exon ‘+’ the position in the intron; if the variant is downstream of the exon, the variant must be identified as the first nucleotide exon ‘−’ the position within the intron.

To ensure that reports communicate laboratory results effectively and meet the user’s needs and best practice guidelines, it is advised that the body of the report include the following:

- Indication for the test/referral reason;
- Interpretation of result and appropriate clinical advice, where possible reports should integrate genetic data with the clinical information that has been provided by the referring clinician;
- Any references cited should be cited in full;
- Information relating to the test undertaken including the test sensitivity, possible limitations and gene panels applied;
- If there were any issues with a sample or sample details.

Clinical reports must also clearly document and identify the laboratory, patient (name, date of birth, NHS number, hospital number and sample number), referring clinician and individual writing the report and the authoriser.

## Genetic counselling (part 2) – post-genetic test input

Once the genetic results have been received, the referring clinician should feed these back to the patient in an appropriate manner. They can offer access to potential therapies if available or refer to the correct multidisciplinary team if there are syndromic features to consider. For example, in patients with congenital cataracts, if a metabolic disease is detected such as cerebrotendinous xanthomatosis caused by mutations in the *CYP27A1* gene resulting in accumulation of cholestanol, replacement therapy with chenodeoxycholic acid normalises plasma levels and improves the neurological and non-neurological symptoms. Patients with biallelic *RPE65* variants causing autosomal recessive *RPE65-*retinopathy are now eligible for the first approved retinal gene therapy called Luxturna or voretigene neparvovec.^131^ This treatment uses a modified adeno-associated virus (AAV) vector, containing human wildtype *RPE65* cDNA under the control of a ubiquitous promoter, which is introduced to the subretinal space through vitrectomy and subretinal injection.^132^ The wildtype/healthy *RPE65* is expressed in RPE cells and leads to improved performance on the multi-luminance mobility test (MLMT) and full-field light sensitivity threshold (FST) up to 4 years, with ongoing monitoring. Where an approved treatment does not exist, there are numerous clinical trials in progress for ocular genetic-based therapies that can be shared with patients.^131^ A useful website to keep abreast of clinical trials relating to a specific gene or condition is ClinicalTrials.gov (https://clinicaltrials.gov). Not all the trials listed are ethically approved and caution must be taken when recommending a study but it can guide advice to patients.

Some genetic results relating to the eye disorder may change a diagnosis or can reveal a risk of other syndromic problems which may not have been evident previously. For example, some ciliopathy gene defects, such as *IQCB1*, can manifest with an IRD, but heralds a significant risk of kidney disease. These patients should be referred to a relevant specialist for management, screening and metabolic tests.^134^ A genetic result can confirm a severe diagnosis and reduced life expectancy, for example, Battens disease (*CLN3*) or Wolfram syndrome (*WFS1*). These cases will require complex management, including ongoing counselling support and referral to the relevant specialist teams.

Genetic counsellors will help patients to interpret and act upon these results, including segregation analysis, family planning options, incidental findings, cope with the emotional and psychological impact. In the event of a ‘positive’ autosomal recessive result where a pathogenic or likely pathogenic cause of a patient’s condition has been identified, segregation analysis may be necessary. Segregation analysis evaluates the transmission of genetic changes within a family. Autosomal recessive results will be either a homozygous pathogenic change or compound heterozygous pathogenic changes. For compound heterozygous results to be meaningful, the two changes need to be on different alleles (in *trans*), but it can be possible that the two mutations are on the same allele (in *cis*). A DNA sample from a relative – usually a parent or child – can be tested for the two mutations and if the relative only carries one, rather than both or neither, it can be confirmed that the variants are in *trans*.

Segregation analysis can also be useful in the interpretation of autosomal dominant results, when a variant of uncertain significance has been found. Results from both affected and unaffected relatives can increase a variant’s likelihood of pathogenicity. The more relatives whose DNA can be tested, the stronger the evidence becomes. Also, for affected relatives, it is more meaningful, the more distant the relationship is from the proband. For segregation of autosomal dominant results, relatives DNA should be tested only for the presence or absence of the suspected variant.

In the case of unsolved or negative (no primary findings) results, a genetic counsellor should discuss these with the patient and explain in clear terms what the reason for the negative result might be. This conversation with the patient could include limitations of technology used; the testing applied; and the current knowledge of causative genes. It should be made clear that the results do not exclude a genetic cause for the patient’s condition. Other available testing should be offered to the patient, if relevant, for example, if they have had a targeted gene panel, WGS may be the next suitable step. If a patient is receiving an unsolved result from whole genome or exome sequencing, it should also be explained that a genetic diagnosis may become available at a later date without further testing, as a result of subsequent genetic discoveries.

## Family planning and reproductive options

Genetic counsellors will be able to help patients with family planning. A pathogenic or likely pathogenic result will enable a genetic counsellor to provide a patient with accurate risks to family members. When it is relevant, the genetic counsellor will also be able to discuss what reproductive options are available to patients or the parents of a patient, and refer them to a specialist clinical genetics unit when necessary. These family planning discussions will vary for each case and will be dependent upon the specific diagnosis, inheritance pattern, religious and cultural beliefs of the family. Options that may be available to patients are as follows:

- Conceiving naturally – Depending upon the severity of the condition, the percentage risk to the child and attitudes towards the diagnosis, parents may decide to conceive a child naturally. Other options may be costly, time-consuming, conflict with a patient’s religious or cultural beliefs or may not be available depending upon their diagnosis and existing family situation. For example in the United Kingdom, preimplantation diagnosis is only available to patients who meet a certain age (<40 years) and who do not currently have an unaffected child (see below).
- Gamete or embryo donation – Some patients or parents of patients decide to receive an egg, sperm or embryo donation which can decrease the risk of passing a condition to their child to a general population risk.
- Preimplantation genetic diagnosis (PGD) – If a patient or the parents of a patient meets certain criteria, PGD is a form of *in vitro* fertilisation where both parents donate sperm and eggs which are fertilised in the laboratory to produce several embryos. These embryos are then tested to see whether they are free from the condition and then the healthy embryo(s) can be implanted into the mother. PGD authorisation differs through different countries. While PGD is regulated by the doctors’ discretion in the United States, PGD law varies in Europe.^135^ In Italy, PGD was authorised for fertile couples who carried inherited diseases from 2015^136^ and in Switzerland, it is legal for specific diseases.^135^ In France, PGD is regulated by *Loi relative de la bioéthique*, from the Agence de la Biomédecine, in 2004, and each use of PGD is examined by a Centre Pluridisciplinaire de Diagnostic Prénatal (CPDPN).^135^ In the United Kingdom, there are strict guidelines for eligibility, including the following:
  ○ The diagnosis in question must be serious and on the approved Human Fertilisation and Embryology Authority (HFEA) list.
  ○ The risk to the child of inheriting the condition must be greater than 10%, which usually means that patients with autosomal recessive conditions are not eligible (unless their partner is known to be a carrier). However, parents of a child with an autosomal recessive condition who are likely to have a 25% risk of having another child with the condition would meet this criterion.
  ○ The couple must not have an unaffected child already.
  ○ Both parents must not have any known fertility problems.
  ○ Both parents must be non-smokers.
  ○ The female parent must have a healthy body mass index (BMI: 19–30) and must be under the age of 40 at the time of treatment.

- Prenatal testing (and termination of pregnancy) – After conceiving a child naturally, it can be possible to screen the foetus to see whether the child is affected with a condition. For some developmental conditions (e.g. anophthalmia), a non-invasive detailed ultrasound can show the affected status of the child. For other conditions, invasive prenatal genetic tests such as chorionic villus sampling or amniocentesis may be required in order to determine the affected status of the child which can carry a small risk of miscarriage. A new non-invasive prenatal testing (NIPT) method is now available using a blood sample from a pregnant mother, this contains cell free DNA (cfDNA) from the placenta that carries the DNA of the foetus. This can be used to test for rare genetic diseases that are caused by single gene variation; the result is definitive and does not need to be confirmed by invasive tests. This is referred to as non-invasive prenatal diagnosis (NIPD).^137^ Parents can use information gained from prenatal testing to decide whether or not to continue a pregnancy.
- Adoption
- To remain childless (or not to have any further children)

## Conclusion

Genetic testing has significantly advanced over the past decade, moving from predominantly the research field to a well-scrutinised and monitored accredited clinical service for patients. There are increasing numbers of known disease-causing genes and variants that can be screened in parallel and at low cost. Diagnostic rates are increasing especially with NGS technologies such as WGS. With existing therapies such as voretigene neparvovec, and many emerging clinical trials investigating the use of gene or mutation-specific approaches such as gene replacement using viral vector delivery, small molecule drugs for nonsense mutation suppression and antisense oligonucleotides for splice modulation,^131,138–140^ a molecular diagnosis is essential for patient eligibility. A positive molecular diagnosis also aids in gathering natural history data on the course of disease experienced by gene-specific cohorts in order to help with prognosis and establish outcome measures for response to treatments. For the patient and family themselves, knowing the cause of their condition can provide much comfort, and it will support their family planning decisions.
